# Draft genome sequences of two *Stenotrophomonas* sp. isolates obtained from the venom of the tarantula *Stichoplastoris elusinus* (Araneae: Theraphosidae)

**DOI:** 10.1128/mra.00077-25

**Published:** 2025-05-20

**Authors:** Juan Carlos Cambronero-Heinrichs, Daniela Wicki-Emmenegger, Noelia Rechnitzer, Carlos Víquez, Max Chavarría

**Affiliations:** 1 Centro Nacional de Innovaciones Biotecnológicas (CENIBiot), CeNAT-CONARE, San José, Costa Rica; 2 Department of Agronomy, Food, Natural resources, Animals and Environment (DAFNAE), University of Padova, Legnaro (PD), Italy; 3 Escuela de Química, Universidad de Costa Rica, San José, Costa Rica; 4 Centro de Investigaciones en Productos Naturales (CIPRONA), Universidad de Costa Rica 27915, San José, Costa Rica; 5 Oficina subregional de Alajuela, Sistema Nacional de Áreas de Conservación (SINAC), Ministerio Ambiente y Energía (MINAE) 621331 , Alajuela, Costa Rica; Indiana University Bloomington, Bloomington, Indiana, USA

**Keywords:** *Stenotrophomonas*, venom, tarantula, *Stichoplastoris*, Theraphosidae

## Abstract

Genome sequences of two *Stenotrophomonas* sp. strains are reported. Isolates were obtained from the venom of the tarantula *Stichoplastoris elusinus* (Araneae: Theraphosidae) and are closely related to the recently described *Stenotrophomonas muris*. The announcement highlights the presence of multiple antibiotic resistance genes and their relevance to veterinary and human health.

## ANNOUNCEMENT

Venomous animals have evolved toxins that target vital systems of prey (1). Following envenomation, complications like wound infections may arise, either from secondary sources or direct bacterial inoculation during bites (2, 3). Recent studies suggested that venoms are not sterile but host viable bacteria resistant to toxins (2). Bacteria of the genus *Stenotrophomonas* have been consistently found in the venom of Theraphosidae tarantulas from Costa Rica and India (2, 4, 5). Although it is unclear whether these bacteria are true associates of the venom, as they are also abundant in the tarantula gut (4, 5), their presence warrants further study. Here, we report genome sequences of two *Stenotrophomonas* sp. isolates from the venom of *Stichoplastoris elusinus* (Araneae: Theraphosidae) from Alajuela, Costa Rica (4).

Venom was extracted via electric shocks and plated on lysogeny broth agar (Sigma-Aldrich, USA) for bacterial isolation (4). Isolates stored in 20% glycerol at –80°C were cultured in lysogeny broth (150 mL) (6) and incubated overnight (180 rpm, 30°C). Genomic DNA was extracted using the cetyltrimethylammonium bromide protocol (7), and its quality and quantity were assessed by gel electrophoresis, a NanoDrop ND-2000 spectrophotometer, and a Qubit 4 fluorometer (Thermo Fisher Scientific, USA). A paired-end library was prepared with the Illumina Nextera XT kit (Illumina, USA) and sequenced on a NovaSeq PE150 system by Novogene Ltd. (Singapore). Raw data quality was evaluated using FastQC v0.11.9 (8), with low-quality reads and adapter sequences filtered via Trimmomatic v0.36 (9). Reads were assembled using SPADES v5.2.0 (10) and polished with Pilon v1.24 (11). Contigs smaller than 200 bp were removed manually. Assembly quality was assessed with QUAST v5.2.0 (12), and genome annotation was performed using the NCBI annotation service (13). The default parameters were used for all software. Genome assembly statistics and annotation features are detailed in Table 1.

**TABLE 1:** Annotation and genome assembly statistics of two strains of *Stenotrophomonas muris* obtained from the venom of the tarantula *Stichoplastoris elusinus* (Araneae: Theraphosidae)

	*Stenotrophomonas* sp. SE4V1	*Stenotrophomonas* sp. SEV51
BioSample accession no	SAMN46223173	SAMN46223174
Assembly accession no.	JBKOOS000000000	JBKOOT000000000
No. of raw reads	12,279,814	11,871,790
Genome size (bp)	4,641,908	4,501,221
No. of contigs	32	28
No. of predicted genes	4,317	4,178
G + C content (%)	66.85	66.97
N50 (bp)	499,905	329,043
Coverage (1×) (%)	100.0	100.0
No. of tRNA	70	68
No. of rRNA	2	2

**Fig 1:**
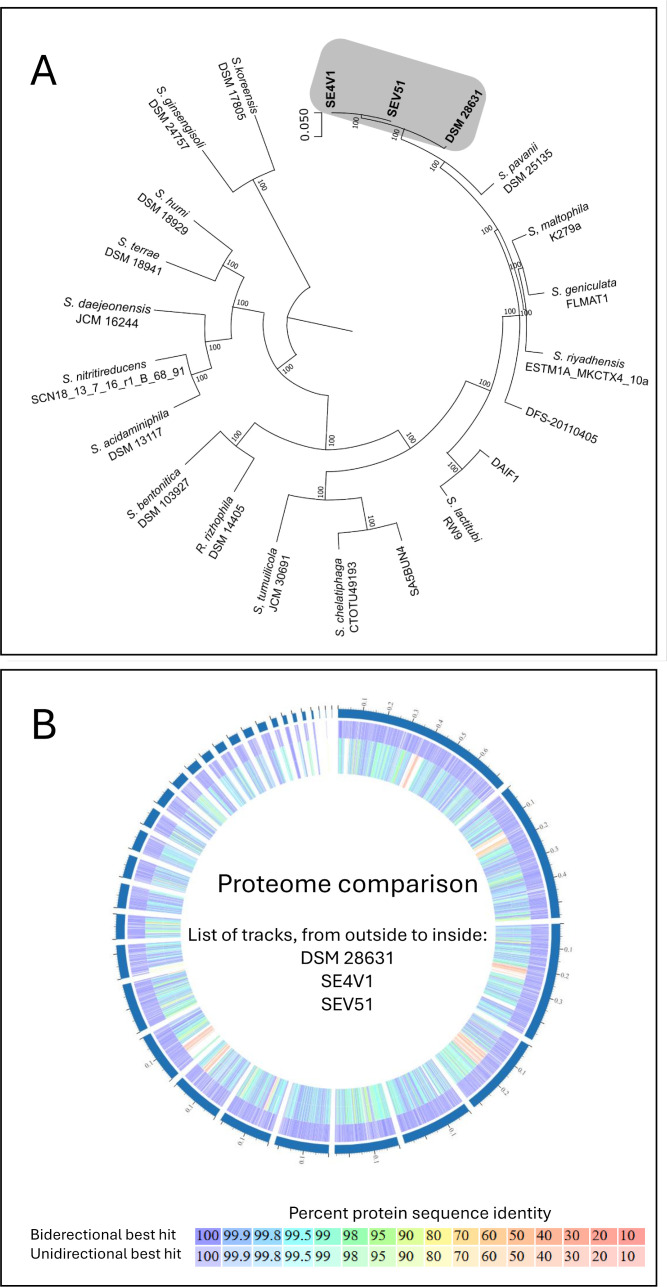
Phylogenomic analysis and proteome comparison of *Stenotrophomonas* sp. strains under study and the reference genome DSM 28631. (A) Phylogenomic tree constructed using 100 genes, showing the isolates SE4V1 and SEV51 (bold) obtained from the venom of the tarantula *Stichoplastoris elusinus* (Araneae: Theraphosidae), along with other strains from the genus *Stenotrophomonas*. Only reference genomes, apart from the strains under study, were included. The closest relative for both sequenced isolates in this study is DSM 28631 (bold), which corresponds to the reference genome of *Stenotrophomonas muris*, a recently described species. Numbers above the branches indicate posterior probabilities, and the scale bar represents the number of substitutions per unit of evolutionary distance. (B) Proteome comparison obtained by PATRIC’s tool (16) between both strains under study and the reference genome DSM 28631.

The phylogenomic analysis conducted on PATRIC identified *Stenotrophomonas* sp. DSM 28631 as the closest relative to both strains (Fig. 1A) (13). Comparative analysis using OrthoANIu (14, 15) revealed similarity values > 98.9% between DSM 28631 and our isolates. PATRIC’s proteome comparison (16) further highlights the distinction between the isolates under study and the reference genome (Fig. 1B). DSM 28631 is the type strain of *Stenotrophomonas muris*, a species recently described in the gut microbiota of mice and found in plant microbiota, rhizosphere, and wastewater (17). It was recently identified as a virulent human pathogen (18). However, according to the List of Prokaryotic Names, *S. muris* is a preferred but not formally correct name (19); we therefore maintained the designation as *Stenotrophomonas* sp. Virulence factors analysis reveals multiple genes in both strains associated with type IV pili but no flagella genes (20). Both strains carry several antibiotic resistance genes, including aminoglycoside phosphotransferases, and multiple efflux pumps (16). The presence of these strains in tarantula venom should be highlighted due to their relevance to veterinary and human health.

## Data Availability

Whole-genome shotgun sequencing for the two microorganisms was submitted to NCBI/GenBank under BioProject PRJNA1209543. Raw reads were deposited with SRA numbers SRR31969038, SRR31969039, and assembled genomes with accessions JBKOOS000000000.1, JBKOOT000000000.1. This paper refers to version 1.0.
